# Associations between genetic variants located in mature microRNAs and risk of lung cancer

**DOI:** 10.18632/oncotarget.9566

**Published:** 2016-05-24

**Authors:** Dengrui Li, Guiyun Zhu, Hongqin Di, Hui Li, Xinyan Liu, Min Zhao, Zhihua Zhang, Yonghui Yang

**Affiliations:** ^1^ Department of General Internal Medicine, Chest Hospital of Hebei Province, Lung Cancer Prevention and Control Center of Hebei Province, Shijiazhuang, Hebei, China, 050041; ^2^ Department of Pathology, Chest Hospital of Hebei Province, Lung Cancer Prevention and Control Center of Hebei Province, Shijiazhuang, Hebei, China, 050041; ^3^ Clinical Laboratory, Chest Hospital of Hebei Province, Lung Cancer Prevention and Control Center of Hebei Province, Shijiazhuang, Hebei, China, 050041; ^4^ Department of Thoracic Surgery, Chest Hospital of Hebei Province, Lung Cancer Prevention and Control Center of Hebei Province, Shijiazhuang, Hebei, China, 050041; ^5^ The First Department of Oncology, Chest Hospital of Hebei Province, Lung Cancer Prevention and Control Center of Hebei Province, Shijiazhuang, Hebei, China, 050041; ^6^ The Second Department of Oncology, Chest Hospital of Hebei Province, Lung Cancer Prevention and Control Center of Hebei Province, Shijiazhuang, Hebei, China, 050041; ^7^ Medical Department, Chest Hospital of Hebei Province, Lung Cancer Prevention and Control Center of Hebei Province, Shijiazhuang, Hebei, China, 050041

**Keywords:** microRNAs, lung cancer, variant

## Abstract

MiRNAs have been focused for their wide range of biological regulatory functions. Previous studies have suggested that individual miRNAs could influence tumorigenesis through their regulation of specific proto-oncogenes and tumor suppressor genes. This study was implemented to investigate the associations between SNPs in mature microRNAs (miRNAs) and development of lung cancer in a two-stage, case-control study, followed by some functional validations. First, 11 SNPs were analyzed in a case-control study of lung cancer, and the significant results were validated in an additional population. Our results showed that rs3746444 in mir-499 (allele C vs T: OR = 1.33; 95% CI = 1.15^−1^.54; *P* = 1.2 × 10^−4^) and rs4919510 in mir-608 (allele G vs C: OR = 1.27; 95% CI= 1.13^−1^.43; *P* = 5.1 × 10^−5^) were significantly associated with increased risk of lung cancer. Rs3746444 in mir-499 was also significantly associated with poor survival of lung cancer (HR, 1.35; 95% CI, 1.15–1.58; *P* = 0.0002). The expression levels of mir-499 and mir-608 were significantly lower than those of adjacent normal tissues (*P* < 0.0005), and the carriers of minor alleles have lower expression levels of mir-499 and mir-608 than those of major alleles (*P* < 0.001). These findings indicated that rs3746444 in mir-499 and rs4919510 in mir-608 might play a substantial role in the susceptibility to lung cancer.

## INTRODUCTION

Lung cancer is the most common cancer in terms of both incidence and mortality worldwide, accounting for 13% of the total cancer cases and 18% of the cancer deaths [1–3]. It's estimated that there will be 0.221 million new cases of lung cancer and 0.158 million deaths in United States in 2015 [4]. According to the National Office for Cancer Prevention and Control in China, lung cancer ranked the third most prevalent cancers among Chinese population [5]. As a complex disease, carcinogenesis of lung cancer is strongly affected by genetic and environmental factors and their complex interactions [6–8].

MicroRNAs (miRNAs) are small (approximately 18-24 nt), noncoding RNAs with important functions in development, cell differentiation, and regulation of cell cycle and apoptosis [9]. They could influence tumorigenesis through their regulation of specific proto-oncogenes and tumor suppressor genes [10–16]. Studies have shown that miRNAs were extremely useful potential agents for clinical diagnostics as well as in personalized care for individual patients [17, 18]. Polymorphisms in the miRNA pathway are emerging as powerful tools to study the cancer biology and have the potential to be used in cancer prognosis and diagnosis, especially for the genetic variants located in the mature miRNAs sequence, which could affect transcription of miRNA primary transcripts and processing of miRNA precursors [19, 20].

In current study, we hypothesized that genetic variants located in the mature miRNAs sequence could influence the susceptibility of lung cancer. Then we systematically searched miRBase (http://www.mirbase.org) for SNPs in miRNAs' mature sequences, and found 11 common (minor allele frequency (MAF) > 0.05) SNPs existing in Chinese population (rs3746444 in mir-499, rs11237828 in mir-5579, rs9295535 in mir-5689, rs12220909 in mir-4293, rs4919510 in mir-608, rs13299349 in mir-3152, rs2168518 in mir-4513, rs10061133 in miR-449b, rs2620381 in mir-627, rs6513497 in mir-646, and rs8078913 in mir-4520a). The hypotheses were tested in a two-stage, case-control study, followed by some functional validations.

## RESULTS

### Demographic variables and clinical information

The selected characteristics of the lung cancer cases and healthy controls in two stages were described in Table 1. The cases and controls were well matched on the distribution of age and gender (all *p* > 0.05). However, Significant differences in smoking status were observed between cases and controls (*p* < 0.001). More than 80% of the histology of the lung cancer patients are non-small-cell lung cancer in both stages.

**Table 1:** Comparison of lung cancer patients and controls by selective characteristics

Variables	Stage I			Stage II		
	Cases (*n* = 500)	Controls (*n* = 500)	*P* value	Cases (*n* = 700)	Controls (*n* = 700)	*P* value
Age (years)	58.2 ± 4.7	58.5 ± 3.9	0.272	60.1 ± 5.4	60.6 ± 4.3	0.056
Gender (male)	391 (78.2%)	389 (77.8%)	0.879	518 (74.0%)	511 (73.0%)	0.672
Smoking status						
Ever	176 (35.2%)	69 (13.7%)	***P* < 0.001**	260 (37.1%)	106 (15.1%)	***P* < 0.001**
Never	324 (64.8%)	431 (86.3%)		440 (62.9%)	594 (84.9%)	
**Histology**						
Small-cell lung cancer	58 (11.6%)			89 (12.7%)		
Non-small-cell lung cancer	442 (88.4%)			611 (87.3%)		
*Adenocarcinoma*	202 (40.4%)			280 (40.0%)		
*Squamous cell*	151 (30.2%)			216 (30.8%)		
*Other NSCLC histology*	89 (17.8%)			115 (16.5%)		

### Association between the mature microRNAs' polymorphisms and risk of lung cancer

In the discovery stage, the genotype distributions and lung cancer risk are presented in Table 2. All of the genotype distribution of these 11 SNPs in controls were in accordance with Hardy Weinberg equilibrium (*p* > 0.05). In the logistic regression analysis, rs3746444 in mir-499 and rs4919510 in mir-608 were independently associated with lung cancer risk after adjusting for age, sex, and smoking status. For the rs3746444 SNP, the C allele conferred 1.37-fold increased risk of lung cancer compared with the T allele (95% CI: 1.09–1.71, *P* = 0.005). Individuals carrying CC genotype had an OR of 1.94 (95 % CI: 1.10–3.40) compared with individuals with TT genotype. While for the rs4919510 SNP, the G allele conferred 1.36-fold increased risk of lung cancer compared with the C allele (95% CI: 1.14–1.63, *P* = 8.02 × 10^−4^). Individuals carrying GG genotype had an OR of 1.78 (95 % CI: 1.24–2.56) compared with individuals with CC genotype.

**Table 2:** Genotype frequencies of candidate SNPs and association with risk of lung cancer in Stage I

Variants	Cases (*n* = 500)	Controls (n=500)	OR^1^ (95% CIs)	*P* value
**rs3746444 in mir-499**				
TT	316 (63.2%)	350 (70.0%)	Reference	
TC	149 (29.8%)	130 (26.0%)	1.27 (0.96–1.68)	0.095
CC	35 (7.0 %)	20 (4.0%)	1.94 (1.10–3.40)	**0.021**
Additive model			1.37 (1.09–1.71)	**0.005**
rs11237828 in mir-5579				
TT	212 (42.4%)	224 (44.8%)	Reference	
TC	199 (39.8%)	211 (42.2%)	0.99 (0.76–1.30)	0.980
CC	89 (17.8%)	65 (13.0%)	1,45 (0.98–2.09)	0.051
Additive model			1.17 (0.97–1.40)	0.093
rs9295535 in mir-5689				
TT	353 (70.6%)	348 (69.6%)	Reference	
TC	114 (22.8%)	133 (26.6%)	0.84 (0.63–1.13)	0.256
CC	33 (6.6%)	19 (3.8%)	1.71 (0.96–3.05)	0.068
Additive model			1.06 (0.84–1.34)	0.597
rs12220909 in mir-4293				
GG	279 (55.8%)	270 (54.0%)	Reference	
GC	181 (36.2%)	195 (39.0%)	0.89 (0.69–1.17)	0.423
CC	40 (8.0%)	35 (7.0%)	1.10 (0.68–1.79)	0.683
Additive model			0.98 (0.80–1.19)	0.839
**rs4919510 in mir-608**				
CC	178 (35.6%)	220 (44.0%)	Reference	
CG	221 (44.2%)	210 (42.0%)	1.30 (0.99–1.71)	**0.049**
GG	101 (20.2%)	70 (14.0%)	1.78 (1.24–2.56)	**0.002**
Additive model			1.36 (1.14–1.63)	**8.02 × 10^−4^**
rs13299349 in mir-3152				
GG	380 (76.0%)	363 (72.6%)	Reference	
GA	106 (21.2%)	120 (24.0%)	0.84 (0.63–1.14)	0.264
AA	14 (2.8%)	17 (3.4%)	0.79 (0.38–1.61)	0.514
Additive model			0.85 (0.66–1.09)	0.203
rs2168518 in mir-4513				
GG	288 (57.6%)	278 (55.6%)	Reference	
GA	191 (38.2%)	200 (40.0%)	0.92 (0.71–1.19)	0.536
AA	21 (4.2%)	22 (4.4%)	0.92 (0.49–1.71)	0.796
Additive model			0.94 (0.77–1.16)	0.564
rs10061133 in miR-449b				
AA	278 (55.6%)	263 (52.6%)	Reference	
GA	182 (36.4%)	191 (38.2%)	0.90 (0.69–1.17)	0.441
GG	40 (8.0%)	46 (9.2%)	0.82 (0.52–1.30)	0.401
Additive model			0.90 (0.74–1.09)	0.292
rs2620381 in mir-627				
AA	335 (67.0%)	350 (70.0%)	Reference	
AC	140 (28.0%)	131 (26.2%)	1.11 (0.84–1.47)	0.443
CC	25 (5.0%)	19 (3.8%)	1.37 (0.74–2.54)	0.309
Additive model			1.15 (0.92–1.45)	0.221
rs6513497 in mir-646				
TT	335 (67.0%)	340 (68.0%)	Reference	
TG	145 (29.0%)	137 (27.4%)	1.07 (0.81–1.42)	0.614
GG	20 (4.0%)	23 (4.6%)	0.88 (0.47–1.64)	0.692
Additive model			1.01 (0.81–1.27)	0.908
rs8078913 in mir-4520a				
TT	234 (46.8%)	233 (46.6%)	Reference	
TC	213 (42.6%)	227 (45.4%)	0.93 (0.72–1.21)	0.609
CC	53 (20.6%)	40 (8.0%)	1.32 (0.84–2.06)	0.225
Additive model			1.06 (0.87–1.28)	0.563

1 adjusted for age, gender, and smoking status.

### Validation of the significant associations in stage II

Then the two SNPs (rs3746444 in mir-499 and rs4919510 in mir-608) was evaluated in an independent dataset (Table 3). The trend was significantly replicated. When merged together, for rs3746444, C allele was significantly associated with a increased lung cancer risk when compared with T allele (OR: 1.33; 95% CI: 1.15–1.54; *P* = 1.2 × 10^−4^). The adjusted OR for the carriers with the CC genotype was 1.8 (95% CI: 1.25–2.60) and for those with the CT genotype was 1.25 (95% CI: 1.04–1.49) compared with the TT genotype. For rs4919510, G allele was significantly associated with an increased lung cancer risk when compared with C allele (OR: 1.27; 95% CI: 1.13-1.43; *P* = 5.1 × 10^−5^). The adjusted OR for the carriers with the GG genotype was 1.57 (95% CI: 1.24–1.99) and for those with the CG genotype was 1.24 (95% CI: 1.04–1.48) compared with the CC genotype. To remove the possible effect modification of smoking status, we also conducted stratified analyses of the two SNPs. As shown in Table 4, the significant trend didn't change materially. To replicate the previous findings, we evaluated the relation between rs3746444 and lung cancer survival. As shown in Table 5, rs3746444 in mir-499 was also significantly associated with poor survival of lung cancer (HR, 1.35; 95% CI, 1.15–1.58; *P* = 0.0002).

**Table 3:** Genotype frequencies of selected SNPs and association with risk of lung cancer in Stage II and the merged results

Variants	Cases (*n* = 700)	Controls (*n* = 700)	OR^1^ (95% CIs)	*P* value
**rs3746444 in mir-499**				
**Stage II**				
TT	461 (65.8%)	500 (71.4%)	Reference	
TC	195 (27.9%)	172 (24.6%)	1.23 (0.97–1.56)	0.080
CC	44 (6.3 %)	28 (4.0%)	1.70 (1.05–2.77)	**0.031**
Additive model			1.30 (1.07–1.58)	**0.007**
**Merged results**				
TT	777 (64.8%)	850 (70.8%)	Reference	
TC	344 (28.7%)	302 (25.2%)	1.25 (1.04–1.49)	**0.018**
CC	79 (6.5%)	48 (4.0%)	1.80 (1.25–2.60)	**0.002**
Additive model			1.33 (1.15–1.54)	1.2 × 10^−4^
**rs4919510 in mir-608**				
**Stage II**				
CC	271 (38.7%)	310 (44.3%)	Reference	
CG	310 (44.3%)	295 (42.2%)	1.20 (0.96–1.51)	0.089
GG	119 (17.0%)	95 (13.5%)	1.43 (1.04–1.96)	**0.025**
Additive model			1.21 (1.04–1.41)	**0.014**
**Merged results**				
CC	449 (37.4%)	530 (44.2%)	Reference	
CG	531 (44.3%)	505 (42.1%)	1.24 (1.04–1.48)	**0.15**
GG	220 (18.3%)	165 (13.7%)	1.57 (1.24–1.99)	1.8 × 10^−4^
Additive model			1.27 (1.13–1.43)	5.1 × 10^−5^

1 adjusted for age, gender, and smoking status.

**Table 4:** Stratified analyses of selected SNPs and association with risk of lung cancer by smoking status

	Smokers				Non-smokers			
SNP	Cases (*n* = 436)	Controls (*n* = 175)	OR^1^ (95% CIs)	*P* value	Cases (*n* = 764)	Controls (*n* = 1025)	OR^1^ (95% CIs)	*P* value
**rs3746444**								
TT	279 (64.0%)	124 (71.0%)	Reference		498 (65.2%)	726 (70.8%)	Reference	
TC	122 (28.0%)	44 (25.0%)	1.23 (0.82–1.84)	0.311	222 (29.1%)	258 (25.2%)	1.25 (1.01–1.55)	**0.036**
CC	35 (8%)	7 (4.0%)	2.22 (0.98–5.04)	0.056	44 (5.7%)	41 (4.0%)	1.56 (1.01–2.42)	**0.045**
Additive model			1.42 (1.03–1.96)	**0.029**			1.28 (1.08–1.52)	**4.4 × 10^−3^**
**rs4919510**								
CC	165 (37.9%)	77 (44.0%)	Reference		284 (37.2%)	453 (44.2%)	Reference	
CG	192 (44.0%)	74 (42.3%)	2.03 (1.33–3.10)	**9.3×10^−4^**	339 (44.4%)	431 (42.0%)	1.25 (1.02–1.54)	**0.031**
GG	79 (18.1%)	24 (13.7%)	1.54 (0.91–2.61)	0.112	141 (18.4%)	141 (13.8%)	1.59 (1.21–2.10)	**8.9 × 10^−4^**
Additive model			1.44 (1.08–1.91)	**0.011**			1.28 (1.12–1.47)	**3.3 × 10^−3^**

1 adjusted for age, and gender.

**Table 5:** Associations between mir-499 rs3746444 and lung cancer survival

Variants	Lung cancer patients	Death	Cox model, adjusted HR (95% CI)^1^	*P* value
**rs3746444 in mir-499**				
TT	769	621	Reference	
TC	340	298	1.22 (1.07–1.39)	
CC	77	69	1.97 (1.19–3.17)	
Additive model			1.35 (1.15–1.58)	**0.0002**

1 The Cox regression analysis was adjusted for age, gender, smoking, stage, surgery, chemotherapy, and radiotherapy status.

### Functional validations of effect mir-499 and in mir-608

**Figure 1:**
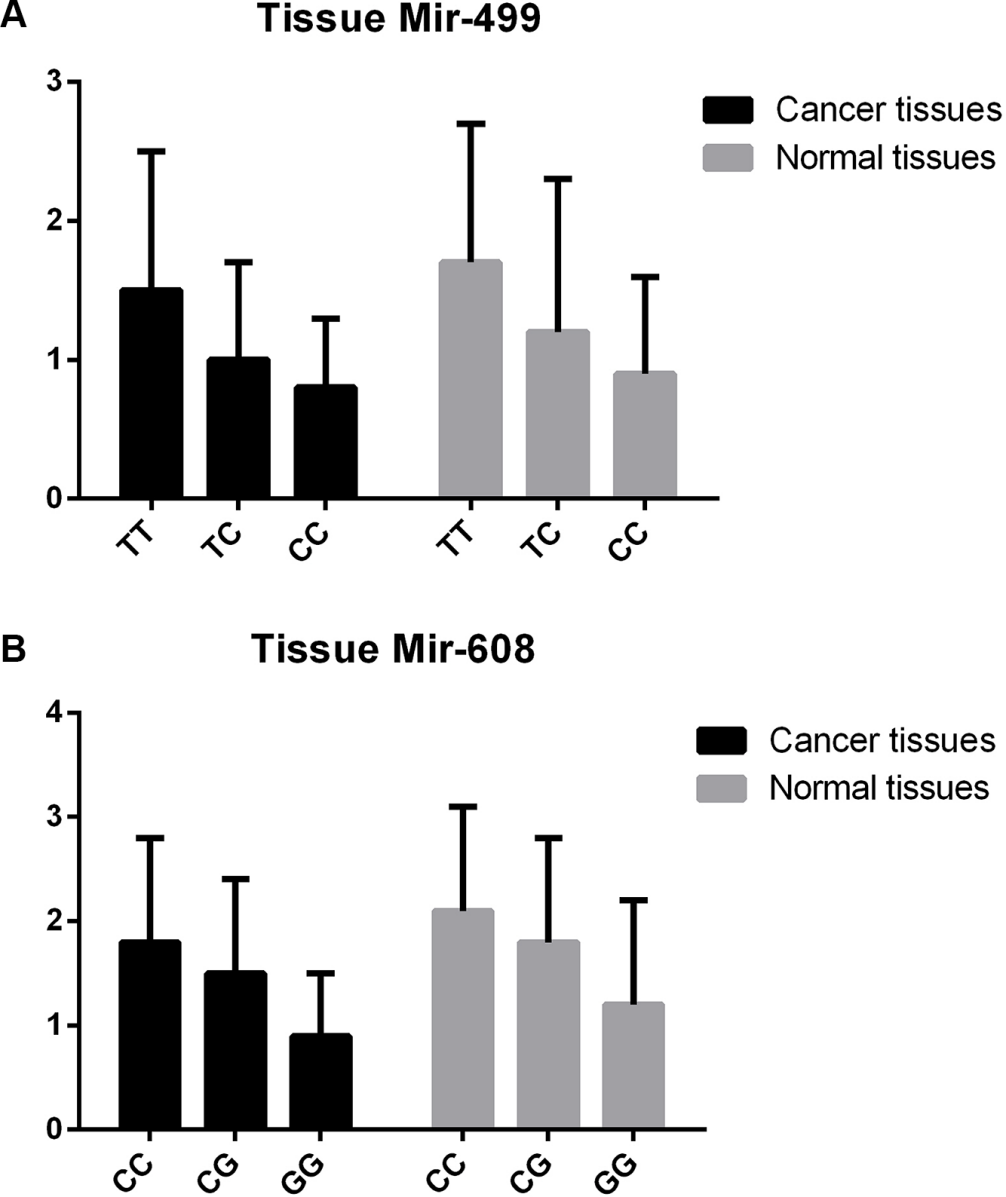
The expression level of mir-499 and mir-608 in lung cancer tissues

**Figure 2:**
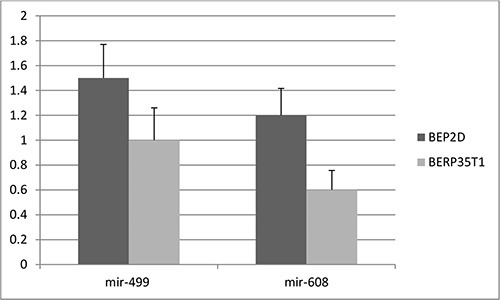
The expression level of mir-499 and mir-608 in cell lines

First, to validate the functional effect of rs3746444 in mir-499 and rs4919510 in mir-608 on corresponding microRNAs in tissues of 500 lung cancer cases, qRT–PCR was used to quantify the expression levels of mir-499 and mir-608 in lung cancer tissues. As shown in Figure 1, the expression levels of mir-499 and mir-608 were significantly lower than those of adjacent normal tissues (*P* < 0.0005), and the carriers of minor alleles have significant lower expression levels of mir-499 and mir-608 than those of major alleles (*P* < 0.001). Furthermore, the expression levels of mir-499 and mir-608 were tested in both the BEP2D cell line and its malignant transformant BERP35T1 cell line. Both mir-499 and mir-608 were down-expressed in BERP35T1, compared with BEP2D cell line (Figure 2).

## DISCUSSION

In this two-stage, case-control study with a total of 1,200 lung cancer cases and 1,200 controls from Han Chinese population, we investigated the associations of 11 common SNPs located in miRNAs' mature sequences with risk of lung cancer. Our results showed that rs3746444 in mir-499 and rs4919510 in mir-608 were significantly associated with increased risk of lung cancer. Rs3746444 in mir-499 was also significantly associated with poor survival of lung cancer. We also found that the expression levels of mir-499 and mir-608 were significantly lower than those of adjacent normal tissues, while the carriers of minor alleles have lower expression levels of mir-499 and mir-608 than those of major alleles. To our knowledge, this is the first study to use multilevel approaches, including genetic association study and gene expression analyses in plasma and tissues, to systematically investigate the effect of genetic variants located in mature microRNAs in relation to etiology of lung cancer.

MicroRNAs have becoming focused circulating biomarkers, given their potential in the translational area and the role of representative readouts of both primary tumor and metastatic deposits [9, 21–23]. Genetic variants located in Mature MicroRNAs have been also explored to discover potential clues for pathogenesis of many diseases [24–30]. Recently, Zhang et al [29] evaluated five SNPs in the mature sequence of microRNAs, and found miR-449b rs10061133 and miR-4293 rs12220909 polymorphisms are associated with decreased esophageal squamous cell carcinoma. Qiu et al [25] evaluated 8 SNPs in the mature sequence of microRNAs, and the results showed that rs4919510 in mature mir-608 sequence is associated with an increased risk of nasopharyngeal carcinoma. Ryan et al [31] found the GG genotype of rs4919510 in mir-608 was associated with an increased risk of death of colorectal cancer. In current study, we also identified rs4919510 in mir-608 was significantly associated with increased risk of lung cancer. Functional validation also confirmed that minor allele G of rs4919510 could increase the expression of mir-608, both in plasma and tissues.

Association of Mir-499 rs3746444 with cancer risk has been fully explored [32–36]. A recent meta-analysis showed that miR-499 rs3746444 polymorphism contributed to increased risk of many cancers (GG versus AA: OR = 1.24, 95% CI: 1.01-1.52; G versus A: OR = 1.11, 95% CI: 1.01–1.23) [32]. This is consistent with our results, which revealed that rs3746444 in mir-499 was significantly associated with increased risk of lung cancer, and the carriers of minor allele C have higher expression levels of mir-499 than those of major allele T. Very recently, Qiu et al [37] also reported that rs3746444 could contribute to poor prognosis by modulating cancer-related genes' expression and thus involve tumorigenesis and anti-chemotherapy. Furthermore, the in silico functional analysis of rs3746444 and rs4919510 was conducted by miRVaS: a tool to predict the impact of genetic variants on miRNAs [38]. The results showed that rs3746444 was located at mature3p(a+5)seed of mir-499. It could change the hairpin structure and affect the mature of the mir-499. While rs4919510 was located at mature5p(a+23) of mir-608, could also change the hairpin structure and affect the mature of the mir-608. All the evidence presented above validated the functions of genetic variants located in mature microRNAs in relation to etiology of cancers.

In summary, our study showed that rs3746444 in mir-499 and rs4919510 in mir-608 could contribute to the carcinogenesis of lung cancer. Although the existence of some limitations, like possible selection bias in case-control study, and limited sample size for interaction analyses, our study still strongly supports the contribution of causative variants located in the mature microRNAs for lung cancer through multilevel approach validations. Further studies with functional and mechanism characterizations, are warranted to provide additional definitive evidence.

## MATERIALS AND METHODS

### Study Subjects

This study consisted of 500 patients with newly diagnosed lung cancer and 500 cancer-free controls in stage I, while 700 lung cancer cases and 700 cancer-free controls in stage II. Patients, which were histopathologically confirmed lung cancer cases, were consecutively recruited between October 2009 and December 2013. While controls were randomly selected from a healthy screening program in the same region and the same population during the same time period as the cases were enrolled. Demographic information were collected for each participant according to a unified procedure. For each participant, approximately 5ml whole blood was obtained to extract genomic DNA for genotyping analysis after the face to face interview. This study conformed to the principles outlined in the Declaration of Helsinki and was approved by appropriate institutional review board. Written consent was obtained from all participants of this study.

### Genotyping

Genomic DNA was extracted from 3-ml of peripheral blood sample using the QIAamp DNA extraction kit (QIAGEN). The candidate SNPs were genotyped using TaqMan real-time polymerase chain reaction (PCR) Assay (Applied Biosystems, Foster city, CA) without the information of the case or control status of the subjects. We used the ABI Prism 7900HT Sequence Detection System analyze the endpoint fluorescence. Quality control was monitored by including 5% duplicate and negative control, with the 100% concurrence rate of the duplicate sets. The average call rate for the candidate SNPs genotyped was > 99.9%. All related primers were provided in Supplemental Table 1.

### Cell culture

The BEP2D cell line (a human papillomavirus 18-immortalized human bronchial epithelial cell line), and The BERP35T1 malignant transformant cell line which were derived from the BEP2D cell line, were cultured to investigate the function of miR-499 and miR-608.

### RNA extraction and Quantification of miRNA by qRT–PCR

Total RNA was extracted from tumor tissues and adjacent normal tissues of 500 cases of lung cancer using Trizol reagent following manufacturer's protocol. Then the amounts of miRNAs were quantified by qRT–PCR using the human TaqMan MicroRNA Assay Kit (Applied Biosystems, Foster City, CA, USA). The expression of miRNAs from tissue samples was normalized using the 2 Ct method relative to U6 small nuclear RNA (RNU6B). The relative gene expression levels were determined using the comparative threshold cycle (2 ^− ΔΔCT^) method.

### Statistical Analysis

Pearson's x^2^ test or *t*-test were used to examine differences between cases and controls in the distribution of demographic characteristics. To evaluate the associations between the genotypes and lung cancer risk, odds ratios (ORs) and 95% confidence intervals (CIs) were calculated by unconditional logistic regression analysis with adjustment for age, gender, and smoking status. The Hardy-Weinberg equilibrium (HWE) for the distribution of each variant was evaluated using the goodness of-fit χ2 test by comparing the observed genotype frequencies with the expected ones in the controls. All statistical analyses were conducted by SPSS v18.0 software, while a two-tailed *P* < 0.05 was used as the criterion of statistical significance.

## SUPPLEMENTARY FIGURES AND TABLES


