# Supplementary material for: Associations between genetic variants located in mature microRNAs and risk of lung cancer
Source: Oncotarget. 2016 May 24;7(27):41715–24. doi: 10.18632/oncotarget.9566 (PMC5173090; doi:10.18632/oncotarget.9566)
Supplement: Supplementary file 1 [file oncotarget-07-41715-s001.pdf]

Associations between genetic variants located in mature microRNAs and risk of lung cancer

Supplementary Materials

1. sequence of miR-499

GCCCTGTCCCCTGTGCCTTGGGCGGGCGGCTGTTAAGACTTGCAGTGATGTTTAACTCCTCTCCACGTGA
ACATCACAGCAAGTCTGTGCTGCTTCCCGTCCCTACGCTGCCTGGGCAGGGT

2. sequence of miR-608

GGGCCAAGGTGGGCCAGGGGTGGTGTTGGGACAGCTCCGTTTAAAAAGGCATCTCCAAGAGCTTCCATCA

AAGGCTGCCTCTTGGTGCAGCACAGGTAGA

Supplemental Table 1: Sequence information for the genes and variants

	Mutations			
Gene Symbol	SNP	location	Forward primer	Reverse primer
mir-499	rs3746444	chr20:34990448	GGCTGTTAAGACTTGCAGTGATGT (forward)	ACGGGAAGCAGCACAGACTT (reverse)
mir-5579	rs11237828	chr11:79422176	TCTGTTCACCTCCACAGTGTTTG(forward)	GGCCCCTGTCACCATTAGCT(reverse)
mir-5689	rs9295535	chr6:10439735	CCTGGACTCTGTCACTTATGTCAATAA (forward)	GCACACACCTGTAGTCCTAGATACTC A(reverse)
mir-4293	rs12220909	chr10:14383222	GGGGAAATCCTATTTCTTCTTCATC (forward)	CCGGAGGTATGGCAGAGACA(reverse)
mir-608	rs4919510	chr10:100975021	TCCCCAGCCCCATTTTCT(forward)	AAGATCCACTGGGCCAAGGT (reverse)
mir-3152	rs13299349	chr9:18573362	TCGTGAACTTCTCACAACAACCTT (forward)	GGGTGGGTGGCTGCAAT (reverse)
mir-4513	rs2168518	chr15:74788737	GTCTCCTGGCCACATTCTAGGT (forward)	GGGCCGAAGTTTTAGACAGCTT (reverse)
miR-449b	rs10061133	chr5:55170716	TCAGGTAGGCAGTGTATTGT (forward)	GGAAAAGCAGGCATAAGTAA (reverse)
mir-627	rs2620381	chr15:42199650	TGGGAGGTGAGGATGAATTG(forward)	GTGGGGCAGAGAGTTTATAG(reverse)
mir-646	rs6513497	chr20:60308547	TCAAGCAGGAAGGGATTCAG (forward)	GGGACTCAGGATGCAAATTG (reverse)
mir-4520a	rs8078913	chr17:6655449	CCTCGCCTGCCTGCTGTA (forward)	CTGCGTGTTTTCTGTCCAAATC (reverse)
